# Magnetic iron oxide nanoparticle enhanced microwave pretreatment for anaerobic digestion of meat industry sludge

**DOI:** 10.1038/s41598-024-61423-6

**Published:** 2024-05-10

**Authors:** Zoltán Péter Jákói, Cecilia Hodúr, Sándor Beszédes

**Affiliations:** Department of Biosystems Engineering, Faculty of Engineering, University of Szeged, Szeged, 6725 Hungary

**Keywords:** Meat industry sludge, Anaerobic digestion, Microwave pretreatment, Iron oxide nanoparticles, Dielectric assessment, Environmental sciences, Environmental biotechnology

## Abstract

Our study investigates the effects of iron oxide (Fe_3_O_4_) nanoparticles combined microwave pretreatment on the anaerobic digestibility and soluble chemical oxygen demand (SCOD) of meat industry sludge. One of our main objectives was to see whether the different microwave-based pretreatment procedures can enhance biogas production by improving the biological availability of organic compounds. Results demonstrated that combining microwave irradiation with magnetic iron oxide nanoparticles considerably increased SCOD (enhancement ratio was above 1.5), the rate of specific biogas production, and the total cumulative specific biogas volume (more than a threefold increment), while having no negative effect on the biomethane content. Furthermore, the assessment of the sludge samples’ dielectric properties (dielectric constant and loss factor measured at the frequency of 500 MHz) showed a strong correlation with SCOD changes (r = 0.9942, R^2^ = 0.99), offering a novel method to evaluate pretreatment efficiency.

## Introduction

The increasing generation of wastewater and sludge due to rapid industrialization and expanding global population growth presents a significant technological and environmental challenge, especially in terms of stabilization and treatment. Conventional sludge treatment methods usually have high cost and energy necessity^1–3^, and therefore alternative, more environmentally friendly, and efficient approaches should be considered. Anaerobic digestion (AD) is a promising biological utilization method for sludge treatment, during which the (soluble) organic compounds are converted into CH_4_ (~ 65%), CO_2_ (~ 30%) and other gaseous components like H_2_S, N, CO, etc. (~ 5%)^4–6^. With the anaerobic fermentation of wastewater sludge, not only the organic load can be reduced, but also the produced biomethane can be used for energetic purposes, as an alternative to natural gas^7,8^. Fermentation residues can also be utilized in soil remediation or fertilizer for example^9–11^; thus, making the AD process a quite complete solution for sludge treatment.

Wastewater sludge generated during different food industry technologies alone or co-digested with food industry waste holds many beneficial properties in terms of biogas production (e.g. high concentration of organic compounds, appropriate water content)^12,13^, and these industries are especially in the need of locally available, alternative energy sources, as their energy demand is usually remarkably high^14–16^, and/or natural gas or other fossil fuels are not easily accessible, particularly in rural areas^17,18^. Since the utilization and preprocessing of meat industry originated sludge for biogas production has not been widely investigated before, in our study we solely focused on the pretreatment and anaerobic fermentation of this biogas feedstock.

Despite its advantageous properties, biogas generation from industrial sludge can sometimes be quite challenging due to different hindering factors^19,20^. Along with the proper C:N:P ratio of the feed^21^, the biological availability of the organic components (i.e. those that are in the soluble phase) plays a major role in biogas yield and quality^22–24^. This means that samples with high soluble chemical oxygen demand (SCOD) usually produce higher quantities of biogas, and thus this parameter, or its proportion to the total chemical oxygen demand (SCOD/TCOD) often used—among others—as an efficiency indicator in AD and/or sludge processing^25,26^. Native (i.e. untreated) sludge samples in many cases do not have a favourable SCOD/TCOD value and thus great (biological) availability^27,28^, because a significant amount of organic components are “locked” or “trapped” inside micro- and macro flocculates, extracellular polymeric substances (EPS) or other solid particles^29,30^. This means that a proper pretreatment step on the sludge feed is usually needed before the biogas fermentation to achieve a satisfactory bioconversion efficiency^31–33^.

Microwave (MW) irradiation has already proved to be an effective pre-processing method in several biomass utilization technologies, as well as in sludge treatment^34–36^. Using microwave pretreatment alone or in combination with other methods on sludge samples can improve several factors and properties, such as dewaterability, disintegration degree, and most importantly the bioavailability of essential organic compounds^37–39^, making the sludge feed more suitable for anaerobic digestion. It is worth mentioning however that the efficiency of microwave pre-processing not only depends on the extent of transmitted MW energy and power, but also on the physicochemical properties of the given substance that is being exposed to it^40,41^. Due to the frequency-dependent molecular and atomic mechanisms behind microwave heating (mainly dipole rotation and ionic conduction), it can be used effectively on those materials that can easily absorb and dissipate electromagnetic energy—in other words, those that have a generally high dielectric constant (ε′) and loss factor (ε″)^42–44^. Besides microwave heating, the change of these—and other—dielectric parameters can be used to control and monitor different utilization processes, as we have already shown in some of our earlier works^45,46^.

**Figure 1:**
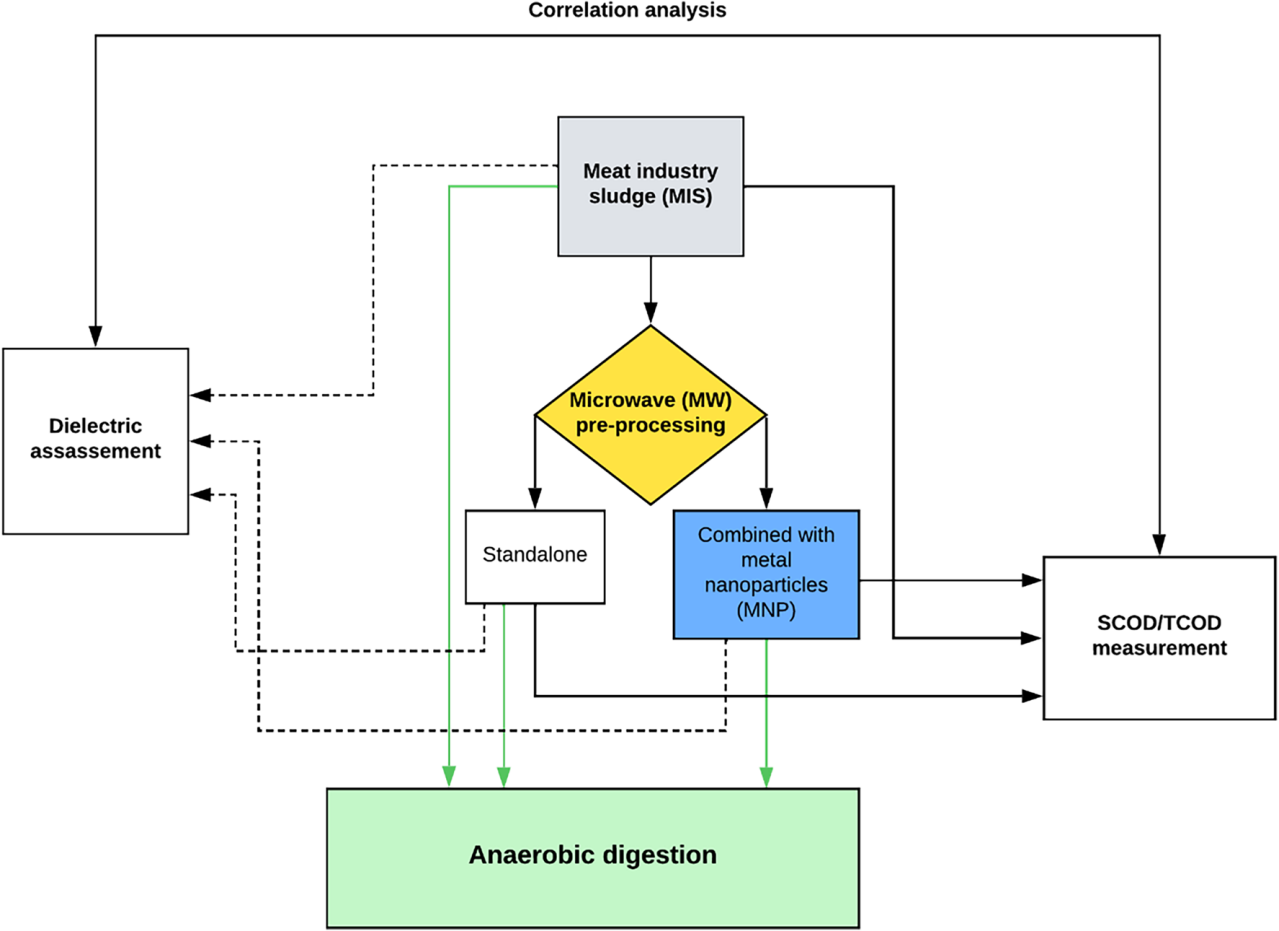
Schematic diagram of the experimental setup.

Material homogeneity is also crucial when it comes to microwave preprocessing techniques; samples that have a homogeneous structure can be heated more evenly in a microwave field as opposed to those that have a heterogeneous composition^47,48^. In terms of wastewater sludge, the potential limiting factors for effective microwave pretreatment are generally the heterogeneous material composition and structure, and the extent of bound water content^49^. Combining microwave treatment with metal nanoparticles (MNPs) has been shown to enhance different chemical reactions^50–52^ as MNPs act as local hot spots due to the selective heating mechanism of microwave radiation. In case of enhancing the anaerobic utilization of any type of sludge, three different options are generally available: applying a pretreatment step on the sludge feed, increasing the microbial metabolism or growth, and optimizing the process parameters of the AD. Out of these three, the use of MNPs might have a positive effect on the former two, i.e. by increasing the efficiency of the pretreatment step, and by contributing to the microbial metabolism in a positive way. Former studies concluded that iron is part of different hydrogenase enzymes that plays a role in H_2_ uptake, as well as of CODH (CO-dehydrogenase), which is a central biocatalyst in the formation of CH_3_COOH—the main forming component during the acetogenesis phase of AD^53,54^. This suggest that the presence of (biologically available) iron can contribute to the proper or increased functioning of these enzymes, which, ultimately, might result in an enhanced bioconversion efficiency during the anaerobic digestion. There is also available research data that shows how magnetic iron oxide nanoparticles behave when put into contact with an electromagnetic (EM) field; demonstrating that using 110.7 kHz frequency is enough to reach approximately 0.12 K/s heating rate with the appropriate conditions^55^. This mechanism is explained by that phenomenon of dielectric relaxation, such that the particles are not able to follow the change of polarity in a rapidly alternating EM field, causing them to ‘lose’ a huge proportion of absorbed EM energy as heat. Microwave equipment, however, operate at higher frequencies (usually 2450 MHz), resulting in considerably higher field strength, and therefore much higher temperatures. This means that if appropriate MNPs are adequately distributed in a heterogeneous feed (like sludge), they can act as local hot-spots and therefore might heat up the material in a more efficient way, resulting in an increased disintegration degree and thus, higher SCOD. Reaching higher SCOD is usually one of the key objectives in terms of sludge pretreatment, which can be achieved in several different ways. Research findings demonstrate that using alkaline treatment, microwave irradiation, the combination of these two, or other physiochemical treatments can effectively increase the SCOD/TCOD ratio of municipal sludge^56,57^, however, relevant experimental data on the pretreatment of industrial sludge in this regard are scarce. In case of sludge treatment, using MNPs to enhance microwave pretreatment, and thus increasing the SCOD has not been investigated before. Therefore, the main objective of our research was to verify whether the addition of magnetic Fe(II,III)-oxide nanoparticles to meat industry sludge feed can improve the efficiency of microwave preprocessing prior to AD by overcoming the limiting factors due to the material inhomogeneity, and such, resulting in greater increment in the bioavailability of organic compounds. Another focal point of the research was to determine if the change occurring in the aforementioned dielectric parameters (dielectric constant and loss factor) of the sludge samples can be used to determine (or estimate) the efficiency of these pre-treatment methods, serving as an alternative technique for conventional analytical procedures like chemical oxygen demand measurement. Figure 1 shows the schematic layout of the applied experimental steps and procedures.

## Methods and materials

The experiments were conducted by using industrial wastewater sludge originated from a local meat processing factory. The basic analytical parameters and characteristics of the native samples are shown in Table 1. Analytical tests were carried out in accordance with the standardized methods for water and wastewater treatment and analytics (APHA standards^58^). Total organic carbon (TOC) and total nitrogen (TN) was determined with a Torch TOC/TN combustion analyser (*Teledyne Tekmar*, USA). For all the experiments, 100 mL of sludge sample was being used, which was supplemented by 10 mL of inoculum sludge prior to the anaerobic digestion to ensure the appropriate microbial composition (the characteristics can be observed in Table 1).

**Table 1:** Analytical parameters of the native (non-treated) meat industry sludge and the mesophilic inoculum sludge.

Parameter	Value + SD	Unit
Meat industry sludge		
pH	6.3 ± 0.2	[–]
Total solids (TS)	14.4 ± 0.6	g/L
Total chemical oxygen demand (TCOD)	34.3 ± 1.1	g/L
Soluble chemical oxygen demand (SCOD)	11.1 ± 0.6	g/L
Biochemical oxygen demand (BOD_5_)	2.79 ± 0.15	g/L
Total organic carbon (TOC)	3.2 ± 0.09	g/L
Total nitrogen (TN)	0.947 ± 0.01	g/L
Mesophilic inoculum sludge		
pH	6.85 ± 0.25	[–]
TS	29 ± 0.25	g/L
TCOD	46.98 ± 1.04	g/L
SCOD	5.15 ± 0.19	g/L
TOC	28.6 ± 1.1	g/L
TN	16.31 ± 0.8	g/L

Microwave pre-treatments were achieved with a Labotron 500 laboratory grade microwave unit, operating at a frequency of 2450 MHz. In contrast to conventional household MW ovens, this unit is equipped with a continuously radiating magnetron, which ensures a homogeneous transmission of the electromagnetic waves. Since earlier scientific literatures reported that the strength of the MW field (which is directly affected by the power of the microwave equipment) influences the overall effectiveness of an MW treatment^59,60^, the experiments were carried out at two power levels: 250 W and 500 W, with a corresponding operational time of 360 s and 180 s, respectively. This means that the samples received 2.5 W/mL and 5 W/mL specific MW power, respectively.

For the MW-MNP combined experiments, magnetic iron oxide nanoparticles (median particle size of 109.9 ± 2.4 nm, point of zero charge (PZC) at pH = 7.9, specific surface area of 95.3 m^2^/g) were added to the samples to achieve a final concentration of 5 mgMNP/100 mL sludge. This concentration was chosen based on our preliminary control experiments. The MNPs were obtained from another research group actively working in our University; the process of synthesis, XRD-identification of the iron oxide product, and the detailed characteristics can be found in their previous papers^55,61^. Prior to the microwave treatments, the MNPs were distributed evenly in the sludge samples with a 30 min stirring (400 rpm). In one part of the experiments the MNPs were kept in the sludge samples during the subsequent anaerobic digestion, while in the other part the nanoparticles were removed from the feed using rapid, multi-stage magnetic stirring. Based on control mass measurements, 99.7–99.8% of the MNPs were successfully removed from the samples in each case. In the case of the former, we abbreviate and refer to these samples as MNPR in the manuscript (as in metal nanoparticles *removed*). To understand the various effects caused by the presence of MNPs in the system to a broader extent, we created an MNP ‘control’ as well, which did not receive any MW treatment, but the nanoparticles were kept in the samples during the AD. The different pre-treatment methods and samples are summarized in Table 2.

**Table 2:** The different pre-treatment methods on the sludge samples.

Sample ID	Microwave treatment (specific MWP)	MNP during MW treatment	MNP during AD
Control	–	−	−
MNP	–	−	+
250 W3 min	250 W, 3 min (2.5 W/mL)	−	−
500 W1.5 min	500 W, 1.5 min (5 W/mL)	−	−
250 W3 min MNP	250 W, 3 min (2.5 W/mL)	+	+
500 W1.5 min MNP	500 W, 1.5 min (5 W/mL)	+	+
250 W3 min MNPR	250 W, 3 min (2.5 W/mL)	+	−
500 W1.5 min MNPR	500 W, 1.5 min (5 W/mL)	+	−

After the different pre-treatment procedures, the subsequent anaerobic digestion took place in laboratory grade borosilicate glass fermenters with a total capacity of 250 mL each. The conditions were set to be mesophilic; the temperature during the process was kept at 38 ± 0.2 °C in a thermostatic cabinet. The samples were stirred continuously for better mass transfer and homogeneity. The pressure difference during the AD was measured with WTW OxiTop IDS/B manometric measuring heads (*Xylem*, USA), and the volume of the produced biogas was calculated via the modified ideal gas law.

To characterize the quality of the produced biogas, the final methane content of the gas samples was measured with an OPTIMA7 biogas analyser (*MRU*, Germany), which is equipped with a pair of NDIR sensor for the detection of CH_4_ (measuring range: 0–100 v/v%, margin of error: ± 0.2 v/v%

**Figure 2:**
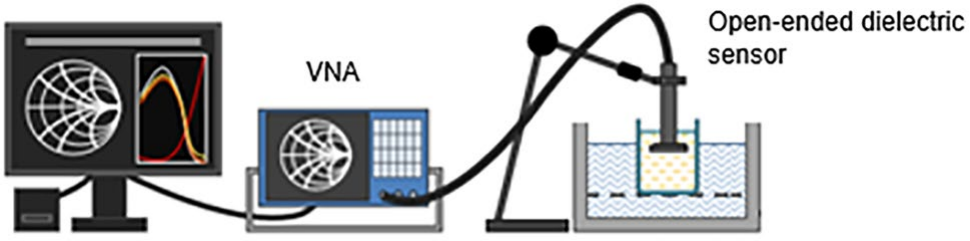
Dielectric assessment kit.

The dielectric assessment of the native (non-treated, i.e. control) and pre-treated samples was carried out with a DAK-3.5 open-ended dielectric probe (*SPEAG*, Switzerland) connected coaxially to a ZVL-3 vector network analyser (VNA), in a frequency range of 200–2400 MHz (Fig. 2). Open-ended dielectric measuring probes are effectively suitable for measuring different biological systems, especially those that are in a liquid phase or in a suspension. When the sensor is immersed in the substance to be measured, the electromagnetic field generated by the coaxial cable interacts with the material. This interaction changes the reflection coefficient of the signal detected by the VNA, in the frequency range corresponding to the measurement. In our experiments we measured the dielectric constant (ε′) and loss factor (ε″) of the soluble phase (supernatant) of the samples. Since these parameters are temperature dependent, each sample was kept at 25 °C during the measurement.

All the different experiments were carried out in three parallels; therefore the experimental results presented are the average values for a given measurement with a corresponding standard deviation (where applicable). The average values of the dielectric parameters were determined and calculated from 30 measured values. The calculations, graphs and figures were created with Microsoft 365 Excel. The numerical results of the dielectric assessment were obtained with the original software of SPEAG DAK (ver. no. 3.6.0.32).

## Results and discussion

**Figure 3:**
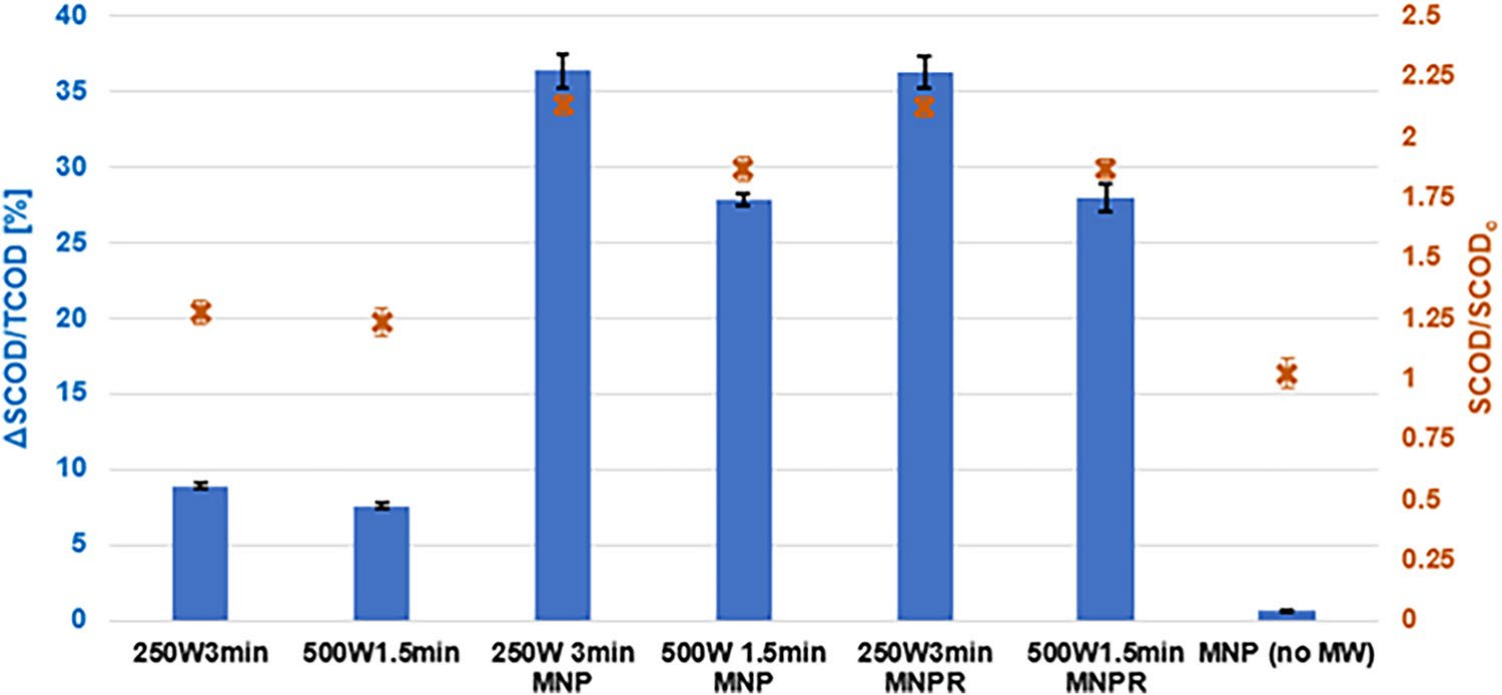
The effect of pre-treatments on the SCOD content of the sludge samples.

In the first part of the experiments, we wanted to see how the different pre-treatment techniques affect the chemical oxygen demand of the samples’ soluble phase (SCOD). To characterize this change, we determined the values for ΔSCOD/TCOD and SCOD/SCOD_c_. The former gives information about how the biologically more accessible, soluble part of the total organic content has changed compared to the control, non-treated sample, while the latter shows exactly how many times the SCOD content of a treated sample is higher (or lower) than that of the control. These results are shown in Fig. 3, on which the left y-axis represents the values of ΔSCOD/TCOD [%], while the right y-axis shows the values of SCOD/SCOD_c_ [–].

It can be clearly seen that the different microwave treatments have caused an observable difference in the soluble chemical oxygen demand, whilst the presence of metal nanoparticles did not alter it in any way (ΔSCOD/TCOD = 0.6%, SCOD/SCOD_c_ = 1.02). The standalone MW treatment resulted in a 7.6–8.9% increment in the SCOD/TCOD value, with the 2.5 W/mL treatment having the slightly stronger effect. Although the difference is clearly not prominent, it might indicate that the applied level of MWP can indeed play a role in terms of sludge disintegration. Using higher power will result in a stronger applied electromagnetic field, which ultimately leads to a steeper heating rate. Since the operational time in case of the 500 W treatment was half of the 250 W one to achieve energy equivalence, the generated heat energy inside the sludge sample might have not been distributed as evenly, causing a slightly smaller degree of disintegration. It was shown in earlier studies as well that applying standalone microwave irradiation with appropriate operational conditions can increase the SCOD/TCOD value of waste activated sludge by even a factor of 10^62,63^.

Combining the microwave treatments with metal nanoparticles undoubtedly has an extensively stronger effect on the values of SCOD. Using 2.5 W/mL MW irradiation coupled with MNPs, the change in SCOD/TCOD was slightly higher than 36%, while the 5 W/mL MW + MNP treatment caused a 27% increase. In terms of SCOD values solely, the combined treatments resulted in SCOD/SCOD_c_ to be 2.13 and 1.87, respectively. Comparing these results to those that of standalone or alkaline-combined microwave heating (aiming at increasing the SCOD ratio) in other available literature, it can be concluded that by using the combination of MW + MNP, the increment in SCOD can be significantly higher—depending on, the raw material, and the applied microwave conditions^64,65^.

Since the presence of MNPs without any other treatment did not have a significant effect on the SCOD, it can be stated that the application of metal nanoparticles could enhance the effects of MW preprocessing on the SCOD content of the sludge. However, the degree of increment depends on the applied level of MW power, just like in the case of the standalone MW treatments. These results suggest that applying a lower level of power with longer operational time will result in a higher efficiency in terms of SCOD increase, indicating that the duration of the microwave irradiation is a more important factor than the effective strength of the applied MW field. Although it is yet to be verified with further experiments directly focusing on the thermal effects and mechanisms of these pretreatment techniques, these experimental findings strongly suggest that the metal nanoparticles acted as local hot spots in the sludge samples, resulting in a higher, more evenly distributed thermal field, which ultimately led to better disintegration and thus, higher SCOD increase^66^.

In case of the samples from which the MNPs were removed after the combined treatment, the results for SCOD/TCOD change and SCOD/SCOD_c_ were basically identical to those in which the nanoparticles were kept, supporting further that the presence of the nanoparticles does not directly affect the concentration of organic matters.

**Figure 4:**
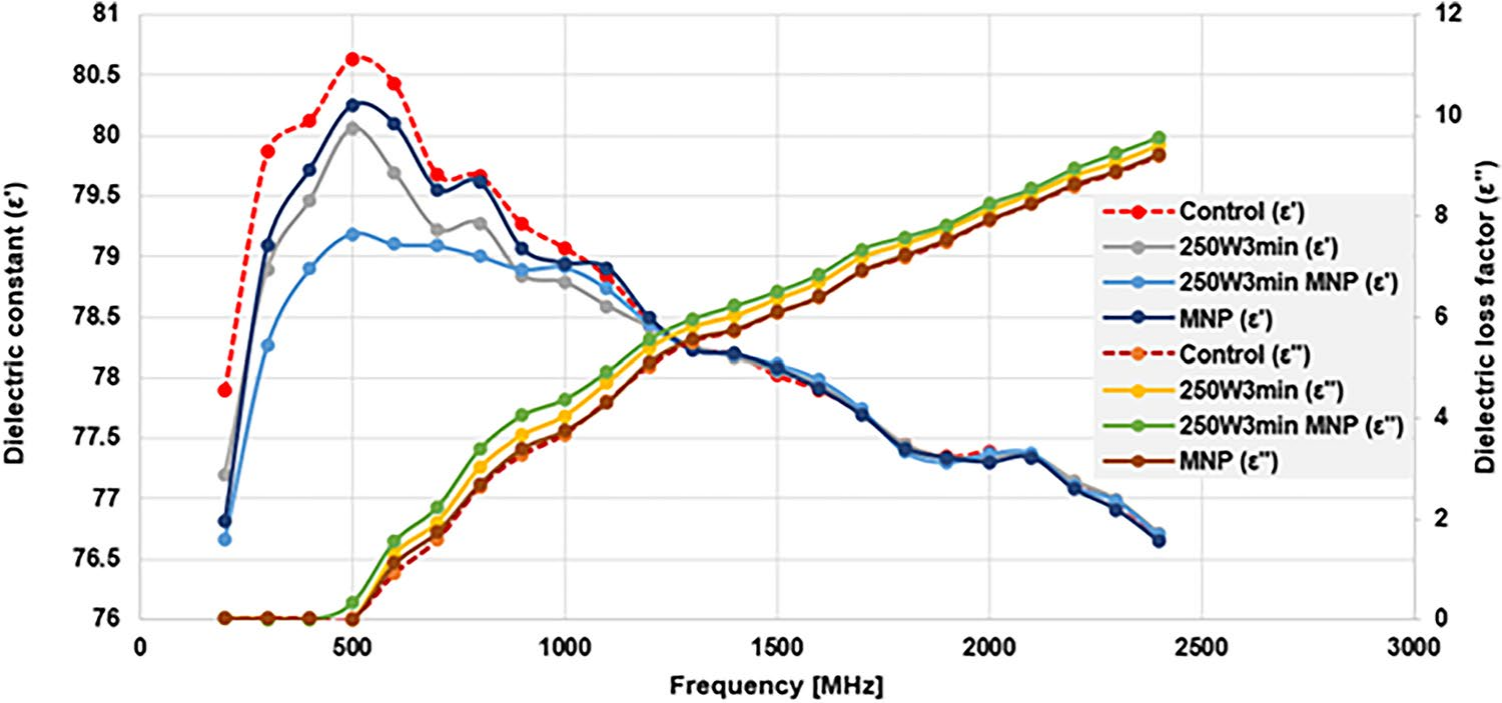
The dielectric spectra of the investigated samples (ε′ and ε″ in f = 200–2400 MHz).

One of our main objectives was to investigate whether the change in the soluble phase of the MIS caused by the different pretreatment methods is reflected by the change in certain dielectric parameters—namely the dielectric constant (ε′) and loss factor (ε″). Figure 4 represents the dielectric spectra (200–2400 MHz) obtained for the control, and some selected pretreated samples.

The most notable differences in the values of dielectric constant occur at the lower frequency range. The reason behind is that most of the soluble phase’s content is water and thus the samples represent a dielectric behaviour surely close to water’s. It is worth noting that the inflection point of the dielectric constant (i.e. the maximal value) occurs at 500 MHz regardless of the type of pretreatment, and in the meantime the corresponding loss factor starts to rise steeply—this proves that neither of the investigated samples show abnormal dielectric behaviour. It is also clear, however, that the effective values of the maximum dielectric constant and loss factor do indeed change depending on the applied pre-treatment method. Compared to the control samples, the most observable change was caused by the MW-treated samples, especially those that were combined with MNPs. These types of treatments have decreased the dielectric constant, meaning that the structural and molecular changes in the investigated material (practically the content of organic materials in the soluble phase) caused its ability to store electromagnetic energy to decrease. Simultaneously, the increased concentration of different organic molecules has caused the dielectric loss factor to increase, which means that these components convert the electromagnetic energy into heat to faster and more easily^42^. The solely presence of MNPs also altered the dielectric behaviour, although to a much smaller extent, as their concentration in the samples was low (5 mgMNP/100 mL). Since the dielectric assessment is a ‘summative’ method, meaning that it gives information about the sample as a whole, the effects of MNPs on the dielectric behaviour of the system was masked by the presence of other, much more abundant components.

**Figure 5:**
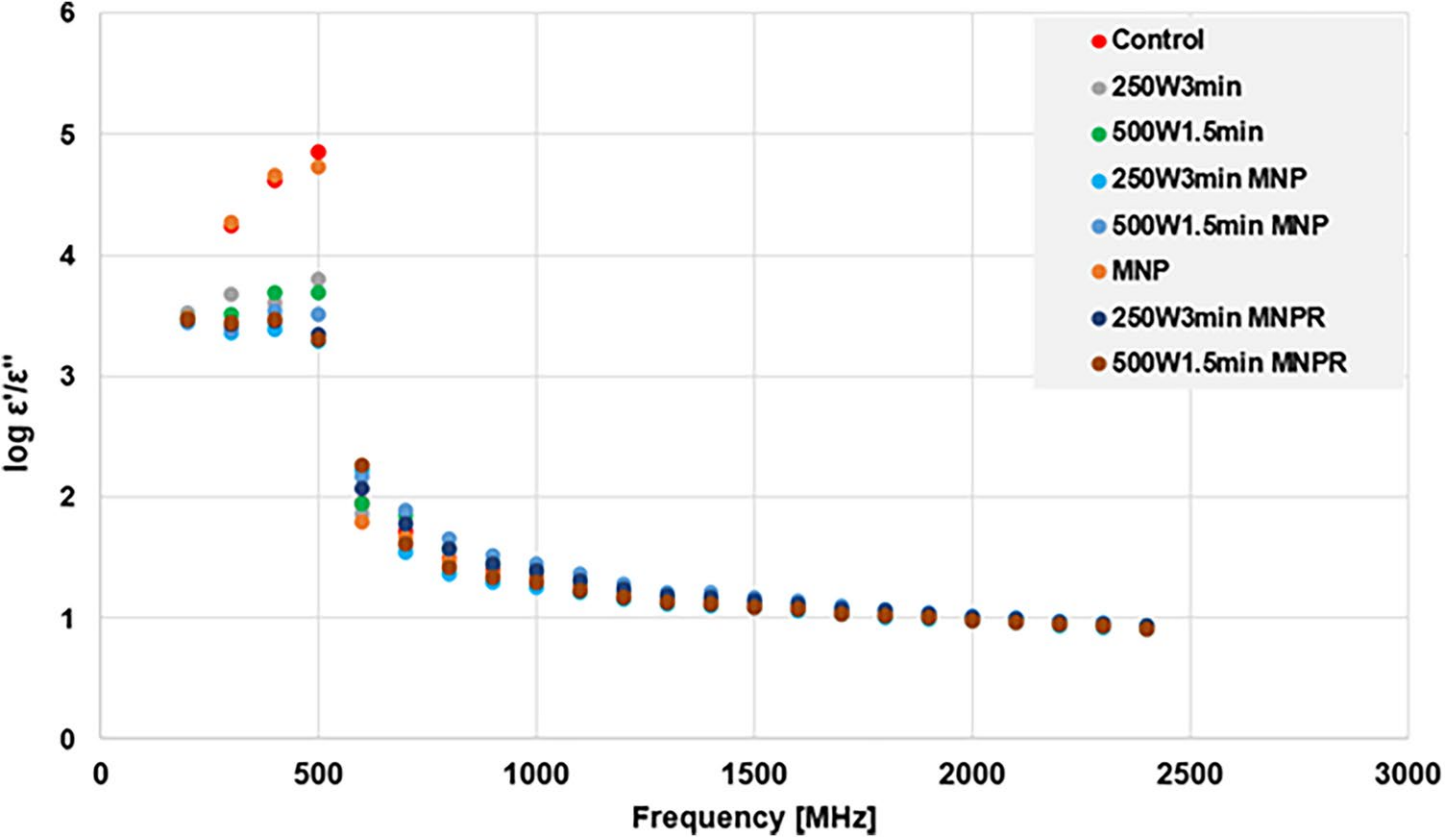
The complex dielectric behaviour of the investigated samples (note: using the semi-log representation makes the comprehensibility, and thus the comparability of the results easier).

The analysis of the dielectric spectrum suggests that the use of pre-treatments on the MIS not only observably influence the SCOD values but also the dielectric behaviour of the investigated material. To verify the extent of this change, it is reasonable to calculate the ratio between the dielectric constant and loss factor (ε′/ε″) of the different samples. If we plot this parameter as a function of frequency, we can analyse and compare the complete dielectric behaviour of these materials simultaneously and see the extent of change the different pretreatment methods caused on them (Fig. 5). This also has the advantage that since there is no proportionality relationship between the change in the dielectric constant and the change in the loss factor (${\varepsilon }^{*}\left(\omega \right)={\varepsilon }^{\prime}(\omega )-i\varepsilon ^{\prime\prime}(\omega )$, where ε* is the complex relative permittivity and ω is the frequency), a change in one parameter does not necessarily mean that the other will change to the same extent.

Based on the results presented on Fig. 5, it can be clearly seen that the use of MW treatment will cause the value of ε′/ε″ to decrease at least an order of magnitude compared to the control samples; and the extent is of it is depending on the exact type of pretreatment used. This is because the loss factor of the MW-treated samples increases significantly in contrast to the non-treated ones, which ultimately reduces the ε′/ε″. This indicates that the disintegration of sludge flocs and other solid components by the microwave treatment considerably changed the material composition of the soluble phase, and the increase in the loss factor implies that these dissolved particles are primarily polarizable and/or ionic in nature. It is also worth mentioning that—regardless of the samples themselves—a distinct drop can be observed at 500 MHz (as suggested by the results on Fig. 4), meaning that the dielectric characteristics of the investigated material matrices starts to ‘converge’ from this point onwards, and the differences are starting to disappear.

**Figure 6:**
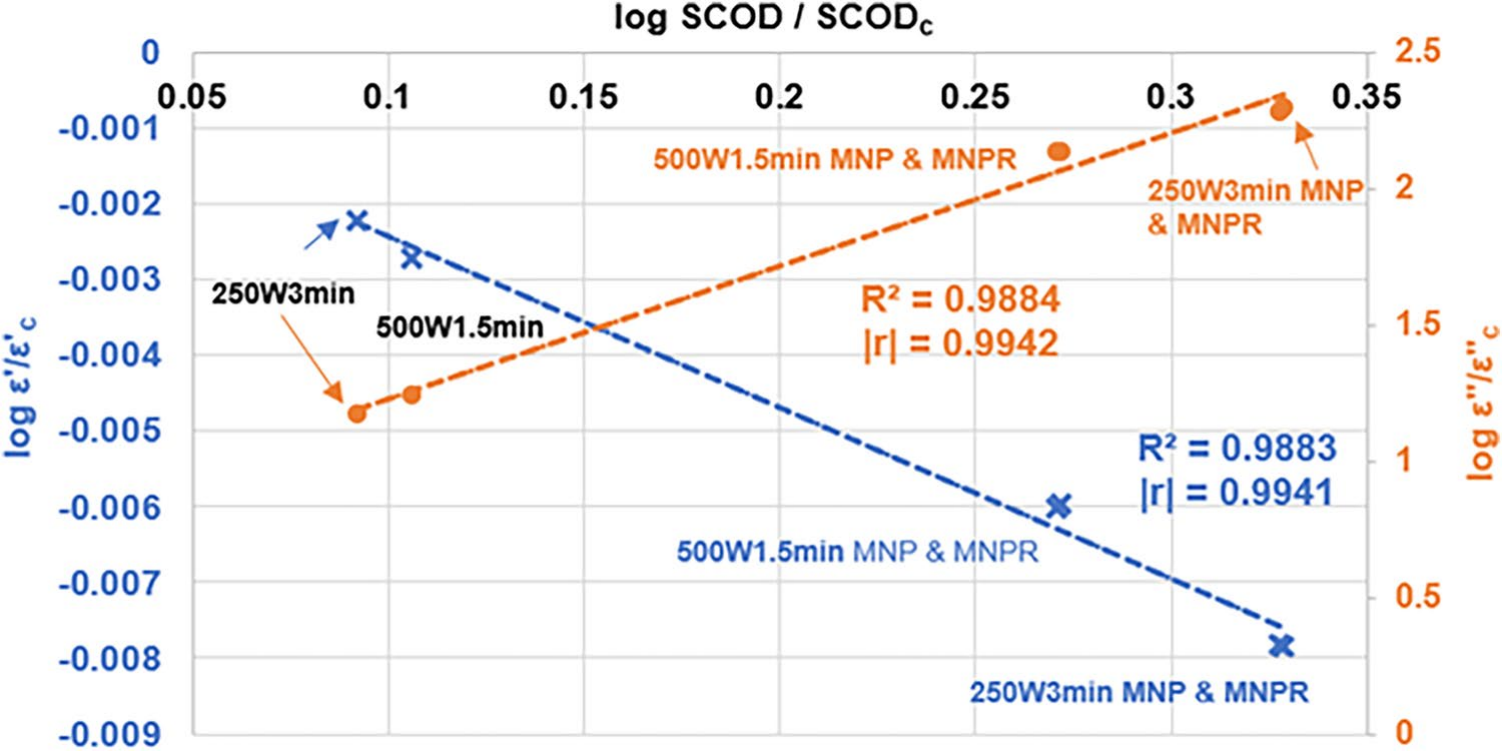
The change in dielectric constant and loss factor (*f* = 500 MHz, T = 25 °C) compared to the control in the function of SCOD change (SCOD/SCOD_c_), in case of the MW-treated samples.

Based on these, during the correlation analysis of the dielectric behaviour and the change in SCOD, the dielectric properties measured at 500 MHz frequency were used (Fig. 6).

Figure 6 shows the results obtained for the change in dielectric parameters versus the change in SCOD caused by the different microwave (standalone or combined with MNPs) treatments on the samples. Since the solely presence of MNPs did not alter either the SCOD or the dielectric parameters of the sludge (Figs. 3, 5), the corresponding results of the MNP control sample were not taken into consideration. For better comparison and to linearize the relationship, we represented these functions with logarithmic transformation. The left y-axis contains the results for the change in the dielectric constant compared to the control, while on the right y-axis the change in loss factor can be observed.

It can be clearly seen from the results for the coefficient of correlation and determination that a strong correlation can be found between the change in dielectric parameters and the change in SCOD generated by the MW preprocessing methods, regardless of the exact type of treatment used (note that points of MW + MNP and MW + MNPR are overlapping, since there was no observable difference between them, regarding SCOD and dielectric properties). This indicates that the relationship between these two parameters is direct, i.e. the molecular change occurring in the soluble phase (SCOD) due to the various pretreatment methods applied will cause the dielectric behaviour to change as well, and the two are obviously closely related. Based upon the r-values of the dielectric constant and loss factor (0.9941 and 0.9942, respectively), it can be stated that measuring either of them can provide accurate information about the organic composition of the soluble phase—which, ultimately, shows us the efficiency of the applied pretreatment process indirectly. Since dielectric assessment is considerably faster and a non-destructive method which does not necessitate the use of chemicals (compared to a conventional chemical oxygen demand measurement), it might provide a reliable alternative to evaluate or estimate the efficacy of different pretreatment techniques.

Although the variations in the feed’s SCOD can predict the outcome of the anaerobic fermentation, it is absolutely necessary to investigate the digestion process of the samples itself to acquire useful and accurate information about the effects of the applied pretreatments. In the second series of the experiments, we used the different sludge samples to generate biogas and investigated the process from various aspects.

One of the important process indicators is the rate of biogas generation or yield, i.e. how much gas (in volume) is formed each day with respect to time. To make the different samples comparable, we normalized this rate to the initial TCOD of the sludge samples (this involves the TCOD of the added inoculum sludge as well). Since there was no observable difference between the initial TCOD values of the different sludge samples, and it has not changed as a result of the treatments, it can be used as an indicator to properly compare the results of the different treatment types. We calculated the specific daily biogas yield as follows:

$${Y}_{b}= \frac{mL}{TCO{D}_{initial}}/day$$

**Figure 7:**
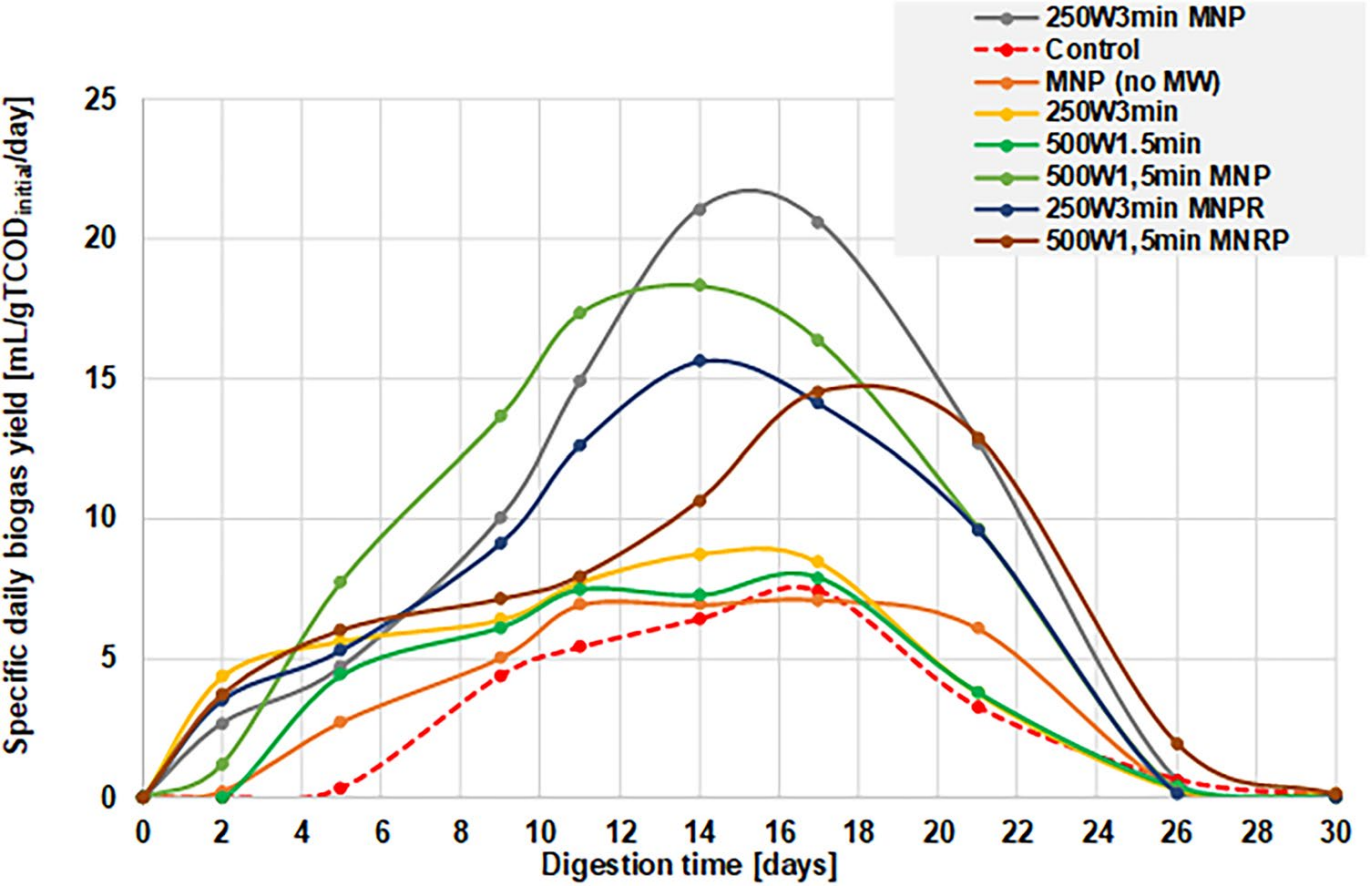
Specific daily biogas yield during the time of AD from the different MIS samples.

This indicator therefore will show how much biogas is generated (in mL) from one gram of the initial TCOD during a 24-h period. The results are shown in Fig. 7.

Based on these results, it can be seen that the peak of the biogas generation (maximum daily yield) is around the 15th day of AD for all samples. Based on the exact value and location of the maximum point, we can acquire information whether a certain pretreatment step can accelerate the biogas formation, and if so, to what extent. It can be observed that the presence of MNP without any MW-treatment did not increase the maximum specific daily yield considerably, however it elongated the biogas generation process, as the rate started to increase earlier (2nd day vs. 5th day), and the decline phase started around the 22nd day, in contrast to the control sample (16-17th day). This suggests that the presence of iron could increase the metabolic activity of certain microbial strains and thus result in increased production, as also reported by Aresta et al.^67^. Although Fe_3_O_4_ cannot be directly utilized biologically, it has been reported that small molecular weight organic acids (such as acetic acid, which is generated during the acetogenesis) can dissolve iron from its oxidized form as ferrous salts, which can be metabolized^61^.

Using standalone MW treatment without MNPs could increase the rate of generation, as well as the maximum specific biogas volume per day, although to a relatively small extent. Comparing the different levels of MWP, using 250 W with 3 min of operational time turned out to be somewhat better than 500 W with 1.5 min (*Y*_*b*_(*max*) = 8.75 mL/gTCOD_initial_/day vs. 7.91), although the difference is not prominent.

Our results verify that the combined pretreatment can not only accelerate the biogas generation throughout the fermentation process, but it can also result in a threefold increase in the maximum specific daily biogas volume (7.2 vs 21.2 mL/gTCOD_initial_/day, in case of the 250 W 3 min MNP sample). The differences between the applied levels of MW power in case of the combined treatments are more apparent than that of the standalone MW treatments, with using 250 W instead of 500 W resulted in higher values for the maximum specific biogas yield (18.3 vs. 21.2 mL/gTCOD_initial_/day). This indicates that in terms of anaerobic digestion the effective irradiation time plays a greater role than the strength of the applied MW field—just like in the case of the SCOD analysis (Fig. 2).

Observing the results obtained for the MW + MNPR samples (from which the nanoparticles were removed prior to AD), we can see that the increment in the specific daily biogas yield falls short to results of the MW + MNP samples, regardless of the applied level of MWP. This suggest that the combination of microwave treatment with metal nanoparticles not only resulted in a more favourable material composition and structure for the anaerobic digestion, but the presence of iron contributed to a higher metabolic activity of certain fermentative microorganisms. This is most possibly due to the fact that iron is part of some enzymes produced by anaerobic bacteria, namely hydrogenases and carbon monoxide dehydrogenase^68^, and therefore the presence of iron is needed for these metabolic pathways to work effectively.

**Figure 8:**
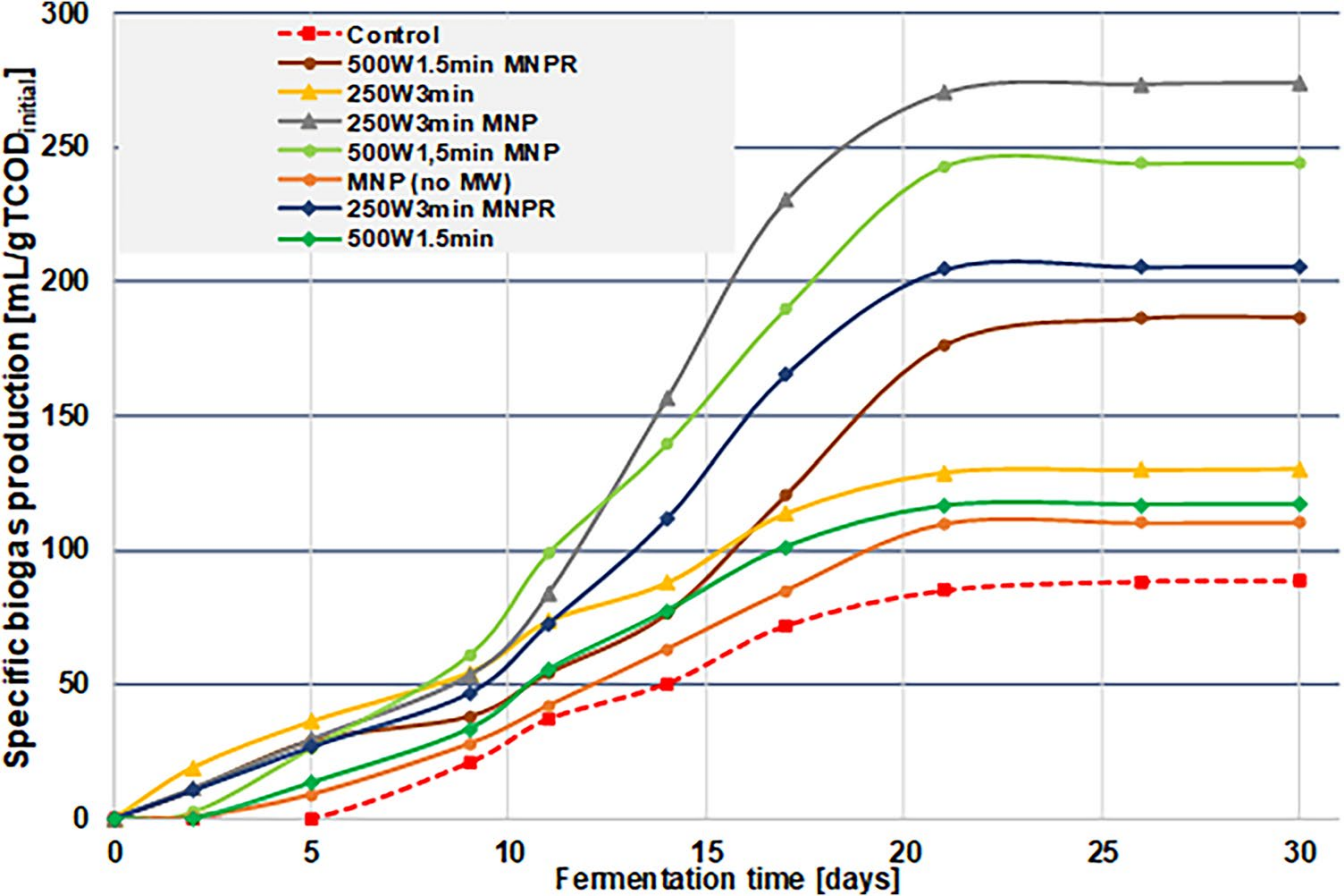
Specific biogas production (cumulative) with respect to fermentation time.

The total cumulative biogas volume with respect to fermentation time can also offer useful and important information about the effectiveness of the pre-treatment techniques, and it provides an opportunity to compare the different samples in a more comprehensive way. Also, it will highlight the different phases (lag, log/exponential, steady-state) of the anaerobic fermentation, which is particularly important from a technological perspective too. Figure 8 represents the specific biogas production in the function of fermentation time for the different (control and treated) MIS samples, i.e. the cumulative biogas volume that can be achieved from 1 g of the initial TCOD of the sludge. Since the initial TCOD of the sludge sample was 34.3 g/L, 100 mL contains around 3.4–3.5 gTCOD plus 0.46–0.47 gTCOD from the addition of 10 mL inoculum sludge, resulting in a total of 3.9–4.0 initial gTCOD. Thus, by multiplying the specific biogas production values with this, we can obtain the total, effective biogas volume approximately.

Results of AD tests show that each of the applied pre-treatment methods could increase the maximum biogas yield compared to the control, however the extent of enhancement varies greatly, depending on the type of pretreatment method used. Standalone 250 W and 500 W microwave treatment without MNP resulted in a maximum specific biogas volume of 117–130 mL/gTCOD_initial_ respectively (in total, effective biogas volume equivalence around 455–515 mL), while the combined MW + MNP pretreatment yielded between 244–278 mL/gTCOD_initial_ (approx. 950–1100 mL in effective biogas volume equivalence) depending on the applied MWP. This means that the combination of 250 W 3 min MW radiation with MNPs resulted in more than a threefold increase in total specific biogas volume compared to the control (88.7 mL/gTCOD_initial_; or around 350 mL in effective biogas volume). Removing the nanoparticles prior to the AD not only led to a decrease in specific biogas production rate, but also in total specific biogas volume (187–205 mL/gTCOD_initial_, or around 730–800 mL effective biogas volume equivalence) compared to the MW + MNP samples—this supports the idea further that MNPs both enhance the effects of microwave treatment and contribute to a more effective microbial metabolism, which ultimately results in a considerably increased biogas yield, compared to other samples. This can also be confirmed by the fact that the solely presence of MNPs without any external energy investment can improve the total biogas volume (110 vs. 88.7 mL/gTCOD_initial_), although to a much smaller extent in contrast to combined methods. These results revealed that using microwave treatment with a combination of metal nanoparticles not only improves the material composition of the sludge feed, but also contributes to a notably enhanced biogas fermentation process. It is also worth mentioning that while the lag phase lasted for approx. 5 days in case of the non-treated sample, the use of pretreatment shortened it to less than 1 day—this might be due to the positive changes in the molecular structure of the soluble phase, i.e. the microorganisms could adapt more easily, when the concentration of dissolved organic compounds was higher^69^.

Although the theoretical maximum biogas yield from unit grams of TCOD is around 600 mL^70^, it is worth noting that in case of wastewater sludge, this value is usually remarkably less (around 250–350 mL/gTCOD), especially for industrial-originated sludge^71^. In our case, the non-treated sample could produce only around 88 mL/gTCOD, which can be explained by the relatively low BOD_5_/TCOD, and the unfavourable C/N ratio. By applying various treatment procedures, we could considerably improve the specific biogas yield (up to almost 280 mL/gTCOD with the MW + MNP method), which further supports the claim that applying appropriate preprocessing techniques can notably enhance the biogas producing potential even for an industry-originated sludge.

**Figure 9:**
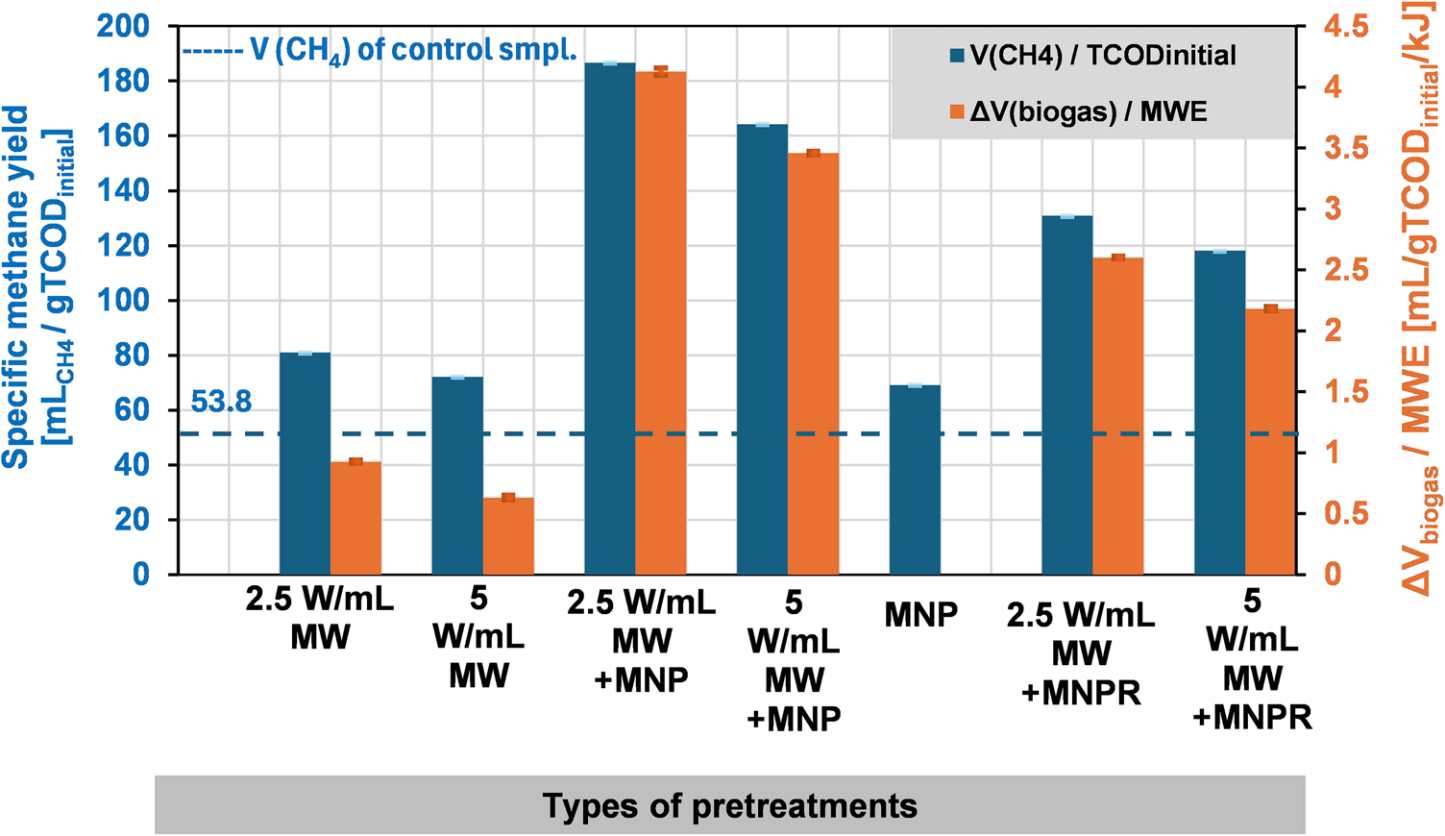
The effects of different pretreatments on biogas quality—specific methane content—(blue), and the increment in specific biogas volume per unit of absorbed MWE (orange). Blue dashed line represents the methane content of the control sample.

To completely evaluate the effectiveness of these pretreatment methods, it is also important to see how these procedures affect the quality of the biogas, i.e. the biomethane content, as well as the amount of utilized MWE with respect to biogas volume. These results are summarized in Fig. 9. The left y-axis (blue) shows the specific methane yield (mL_CH4_/gTCOD_initial_) with the blue dashed line representing the methane content of the control sample, while the right y-axis (orange) shows the change in specific biogas volume caused by a unit of absorbed MWE (mL/gTCOD_initial_/kJ).

It is clearly apparent and important that none of the applied pretreatment techniques had a negative impact on biomethane content; in fact, all of them could improve it to some extent. Standalone MW-treatments without the addition of MNPs resulted in a specific methane yield of 72–81 mL_CH4_/gTCOD, which means that the biomethane content of the produced biogas reached around 61–62% (cf. 53.8 mL_CH4_/gTCOD_initial_; 60% for the control sample). As for the combined MW + MNP treatments, the specific methane volume reached 164–187 mL_CH4_/gTCOD_initial_, which corresponds to a 67–68% methane content. This suggest that using MNP-coupled MW irradiation, not only the overall volume of the biogas can be notably increased, but the quality of it as well. Removing MNPs prior to the AD process resulted in reduced values for the specific methane yield compared to the MW + MNP experiments (118–130 mL_CH4_/gTCOD_initial_; 63–63.5%), which strongly suggests the idea that the addition of MNPs can not only increase the effectiveness of the MW pre-treatment itself, but the presence of iron can also improve the anaerobic digestion process too, making the effects of these two additive. This can be supported further by the fact that the solely presence of MNP (without MW treatment) can also increase the specific methane content as well (69.2 mL_CH4_/gTCOD_initial_; 62.5%). These results also suggest that although the MNPs can be easily separated from the samples before the fermentation, it is not recommended, unless technologically or operation-wise relevant / important.

Regarding the energetic evaluation, these results also suggest that from an energetic point of view, the combined treatments are considered the most effective. Using 2.5 W/mL specific MW power with 3 min of irradiation time in a combination with MNPs results in approx. 4.25 mL/gTCOD_initial_ biogas surplus per unit MWE energy, i.e. 1 kJ of absorbed MWE will produce 4.25 mL more biogas per gTCOD. This increment, however, when using standalone MW treatment is only 0.3 mL/gTCOD_initial_. It is also worth noting that during batch, lab-scale microwave treatments a major portion of irradiated MWE is not utilized, due to volumetric and geometric restrictions of the microwave resonance cavity, therefore in order to effectively investigate the technological and economic feasibility of this method, numerous scale-up experiments are needed, favourably in a continuous microwave system.

## Conclusions

Our study aimed to investigate the effects of microwave treatment with a combination of magnetic iron oxide nanoparticles on the soluble chemical oxygen demand and biogas production of meat industry sludge. We also wanted to see whether the dielectric assessment of the treated samples can be used to determine the effectiveness of the applied pre-treatment methods.

Results revealed that the most considerable increment in the SCOD values can be obtained when combining MW treatment with MNPs, achieving a 35% higher SCOD/TCOD ratio compared to the control sample. This can be explained by the behaviour of MNPs under the influence of an electromagnetic field, namely that due to dielectric relaxation mechanism, they dissipate a huge portion of the absorbed EM energy into heat. Since these iron nanoparticles are distributed evenly in the sludge feed, the effect of microwave heating can be increased and more homogeneous, resulting in a higher degree of disintegration, and thus, higher SCOD.

Based on the measurement of dielectric properties, we found a strong correlation between the change in both dielectric constant and loss factor and the change in SCOD caused by the different pretreatment methods (r = 0.9942 and 0.9941, respectively). This suggest that the change in the physicochemical structure of a sludge feed (i.e. SCOD/TCOD in our case) are reflected in the change of certain dielectric characteristics, providing a promising alternative to evaluate the efficacy of different pretreatment methods on sludge samples.

Regarding the process of anaerobic fermentation, our results indicate that using combined MW + MNP treatment (especially 2.5 W/mL MWP with 3 min of irradiation time) results in the highest cumulative specific biogas yield, and also the uppermost rate of biogas production (278 mL/gTCOD_initial_; 21.2 mL/gTCOD_initial_/day, respectively). This can be explained by the fact that these combined treatments have caused the greatest increment in the SCOD, providing the most (biologically) available compounds to the fermentation microorganisms. Also, as other previous studies have suggested, the presence of iron can contribute to the metabolic activity of certain microbes in a positive way, making the whole AD process more effective.

Future experiments have to focus on the investigation of these presented processes and mechanisms in the case of other types of wastewater sludge (municipal, waste activated sludge, other industrial sludge, etc.) to achieve a deeper understanding, and to identify the possible limitations of these methods. Also, to establish an adequately optimized, effective, and economically feasible preprocessing technique which is based on the combined use of MNPs and microwave radiation, experiments aiming at scale-up possibilities, the use of other available MW frequencies, and the investigation of detailed molecular mechanisms behind MNPs need to be conducted.

## Data Availability

The datasets used and/or analysed during the current study available from the corresponding author on reasonable request.

## Abbreviations

*ε*′: Dielectric constant
*ε*″: Loss factor
${\varepsilon ^{\prime}_c}$: Dielectric constant of the control sample
${\varepsilon ^{\prime\prime}_c}$: Loss factor of the control sample
AD: Anaerobic digestion
BOD_5_: 5-Day biochemical oxygen demand
COD: Chemical oxygen demand
EM: Electromagnetic
MIS: Meat processing industry sludge
MNP: Metal nanoparticle
MNPR: Metal nanoparticles removed (a specific set of samples, from which the MNP was removed prior to AD)
MW: Microwave
MWE: Microwave energy
MWP: Microwave power
SCOD: Soluble chemical oxygen demand (COD of the soluble phase)
SCOD_c_: SCOD of the control sample
TCOD: Total chemical oxygen demand
TCOD_initial_: The initial TCOD of the sludge samples, involving the addition of the inoculum sludge as well
TN: Total nitrogen
TOC: Total organic carbon
*V*_*b*_: Biogas volume
*Y*_*b*_: Specific daily biogas yield (mL biogas produced from 1 g of the initial TCOD during a 24-h period)
